# Generating a Tolerogenic Cell Therapy Knowledge Graph from Literature

**DOI:** 10.3389/fimmu.2017.01656

**Published:** 2017-11-29

**Authors:** Andre Lamurias, João D. Ferreira, Luka A. Clarke, Francisco M. Couto

**Affiliations:** ^1^LaSIGE, Faculdade de Ciências, Universidade de Lisboa, Lisboa, Portugal; ^2^BioISI: Biosystems & Integrative Sciences Institute, Faculdade de Ciências, Universidade de Lisboa, Lisboa, Portugal

**Keywords:** tolerogenic therapy, text mining, knowledge graph, cytokines, machine learning

## Abstract

Tolerogenic cell therapies provide an alternative to conventional immunosuppressive treatments of autoimmune disease and address, among other goals, the rejection of organ or stem cell transplants. Since various methodologies can be followed to develop tolerogenic therapies, it is important to be aware and up to date on all available studies that may be relevant to their improvement. Recently, knowledge graphs have been proposed to link various sources of information, using text mining techniques. Knowledge graphs facilitate the automatic retrieval of information about the topics represented in the graph. The objective of this work was to automatically generate a knowledge graph for tolerogenic cell therapy from biomedical literature. We developed a system, ICRel, based on machine learning to extract relations between cells and cytokines from abstracts. Our system retrieves related documents from PubMed, annotates each abstract with cell and cytokine named entities, generates the possible combinations of cell–cytokine pairs cooccurring in the same sentence, and identifies meaningful relations between cells and cytokines. The extracted relations were used to generate a knowledge graph, where each edge was supported by one or more documents. We obtained a graph containing 647 cell–cytokine relations, based on 3,264 abstracts. The modules of ICRel were evaluated with cross-validation and manual evaluation of the relations extracted. The relation extraction module obtained an F-measure of 0.789 in a reference database, while the manual evaluation obtained an accuracy of 0.615. Even though the knowledge graph is based on information that was already published in other articles about immunology, the system we present is more efficient than the laborious task of manually reading all the literature to find indirect or implicit relations. The ICRel graph will help experts identify implicit relations that may not be evident in published studies.

## 1 Introduction

Tolerogenic cell therapies provide an alternative to conventional immunosuppressive treatments of autoimmune disease and address, among other goals, the rejection of organ or stem cell transplants (1). These therapies aim at modulating the pathological immune response with minimal effect on the immune system. Antigen-presenting cells (APCs) can be induced to control the immune response by targeting specific T cell responses, avoiding general suppression of the immune system (2). It is necessary to understand the underlying mechanisms of the immune system to develop tolerogenic cell therapies. Cytokines are small peptides involved in cell signaling, which can be used to induce tolerance in APCs (3). Immune cells express cytokines and their respective receptors. High-throughput sequencing techniques have improved our knowledge about cell signaling, introducing a variety of information about how cytokines are used by the immune system. This information is important to understand and develop new methods to isolate, culture, and induce tolerance in APCs.

Biomedical information is often presented to the community through published literature, including information about human autoimmune diseases and therapies to treat them. There are knowledge bases aiming at organizing the findings provided by the literature through a single access point. Populating such knowledge bases is, therefore, important for biomedical research, in particular, because they allow computational methods to find patterns in the data, thus generating new hypotheses to be tested experimentally. If a cell produces the same cytokine receptors as another cell, and a new cytokine is found to interact with the first cell, it is plausible that new cytokine could also affect the second cell. This type of inference, also known as ABC model (4), is only possible if the results of many studies are analyzed together.

The scientific community has shown interest in curating databases about cells and cytokines. For example, the National Center for Biotechnology Information (NCBI) provides a compilation of several biomedical and genomic resources (5), including the Entrez Gene database (6). This database contains entries for the genes associated with cytokines, and each entry contains useful information about that cytokine, such as interactions, pathways, and gene ontology annotations. There are also resources specific to cytokine information. The Cytokine Reference is an online database of information on cytokines and receptors, compiled from the literature by experts (7). This database contains links to other databases such as MEDLINE and GenBank, and can be searched by cytokine, cell or disease. Another relevant database is the Cytokine & Cells Online Pathfinder Encyclopedia (COPE)^1^, which focuses on the interactions between cell types through cytokines. The current version of COPE contains 45k entries, including a cell type dictionary of 3k entries. These efforts show the importance of information structures for cells and cytokines. Therefore, the development of computational methods to structure this information would benefit researchers working in this domain.

These computational methods require two conditions: (i) the information is readable by computers and (ii) it is comprehensive, encoding the up-to-date collective knowledge of the community. Both these tasks are currently subject to intensive research. Converting heterogeneous data formats to a common language and merging the data is one approach to the first task. For example, Bio2RDF converts heterogeneous data from several datasets into RDF, a standard data model based on the specification of links between data elements (8).

As for the second task, the information stored in many biomedical datasets is the result of manual processing of documents, which is becoming less practical, since the number of published documents increases at a high rate. A more feasible approach is to use automatic text mining methods to process documents and generate a knowledge graph for a given topic. In a knowledge graph, nodes correspond to real world entities while edges represent relationships between the entities. A widely popular knowledge graph is the one integrated with Google search. This graph is generated from web documents, and organizes information about various topics, such as people, places, and works of art, to improve the quality of the search results delivered to the users.^2^ Recent works have demonstrated how biological knowledge graphs can be extracted from documents, based on protein–protein (9), miRNA–gene (10), and drug–target interactions (11). While these graphs provide important efforts to link the discoveries of various manuscripts, there is still a need for automatic methods that can create specialized graphs and update them as more works are published.

This manuscript presents the system, Identifying Cellular Relations (ICRel), that we developed, based on machine learning, to extract cell–cytokine relations from documents and generate a knowledge graph. ICRel was trained and evaluated with the immuneXpresso database to extract meaningful relations between cells and cytokines in documents. We did not aim at finding novel information, instead we demonstrate the utility of the system by studying the graph generated by ICRel, in particular, the nodes associated with APCs. Therefore, the contributions of this manuscript are: (i) the open source ICRel system that generates a cell–cytokine graph from biomedical abstracts and (ii) the knowledge graph obtained using ICRel on a set of documents relevant to tolerogenic antigen-presenting cell therapy. ICRel was able to identify cytokines associated with tolerogenic antigen presenting cells that were missing from the immuneXpresso database. The code and results obtained with ICRel are available at https://github.com/lasigeBioTM/ICRel.

## 2 Materials and Methods

**Figure 1:**
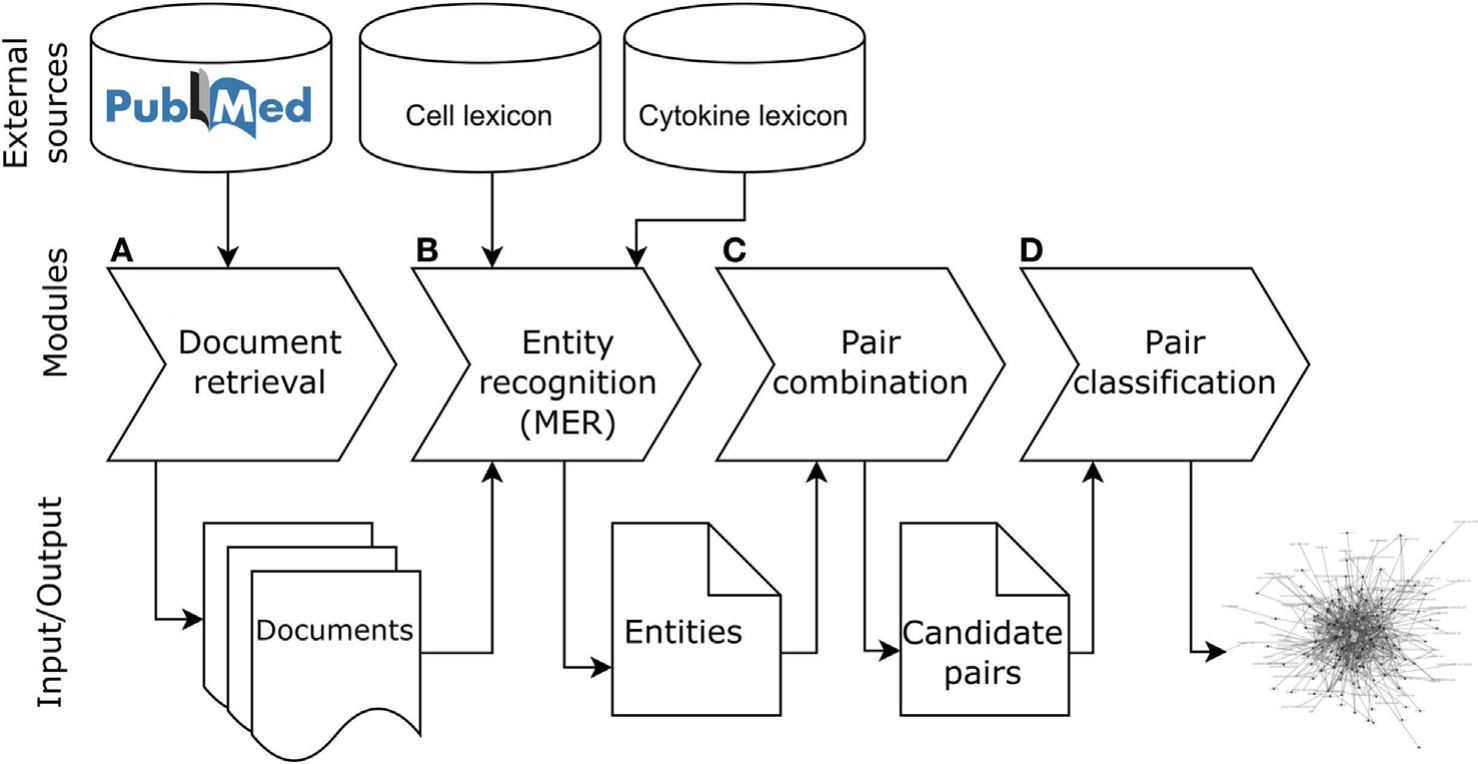
Pipeline of the ICRel system. This first module **(A)** retrieves documents from PubMed, the second module **(B)** annotates cell and cytokine entities in each document using the Cell Ontology and Cytokine registry, the third module **(C)** combines the cells and cytokines mentioned in the sentence, and the fourth module **(D)** classifies each pair and generates the graph.

**Figure 2:**
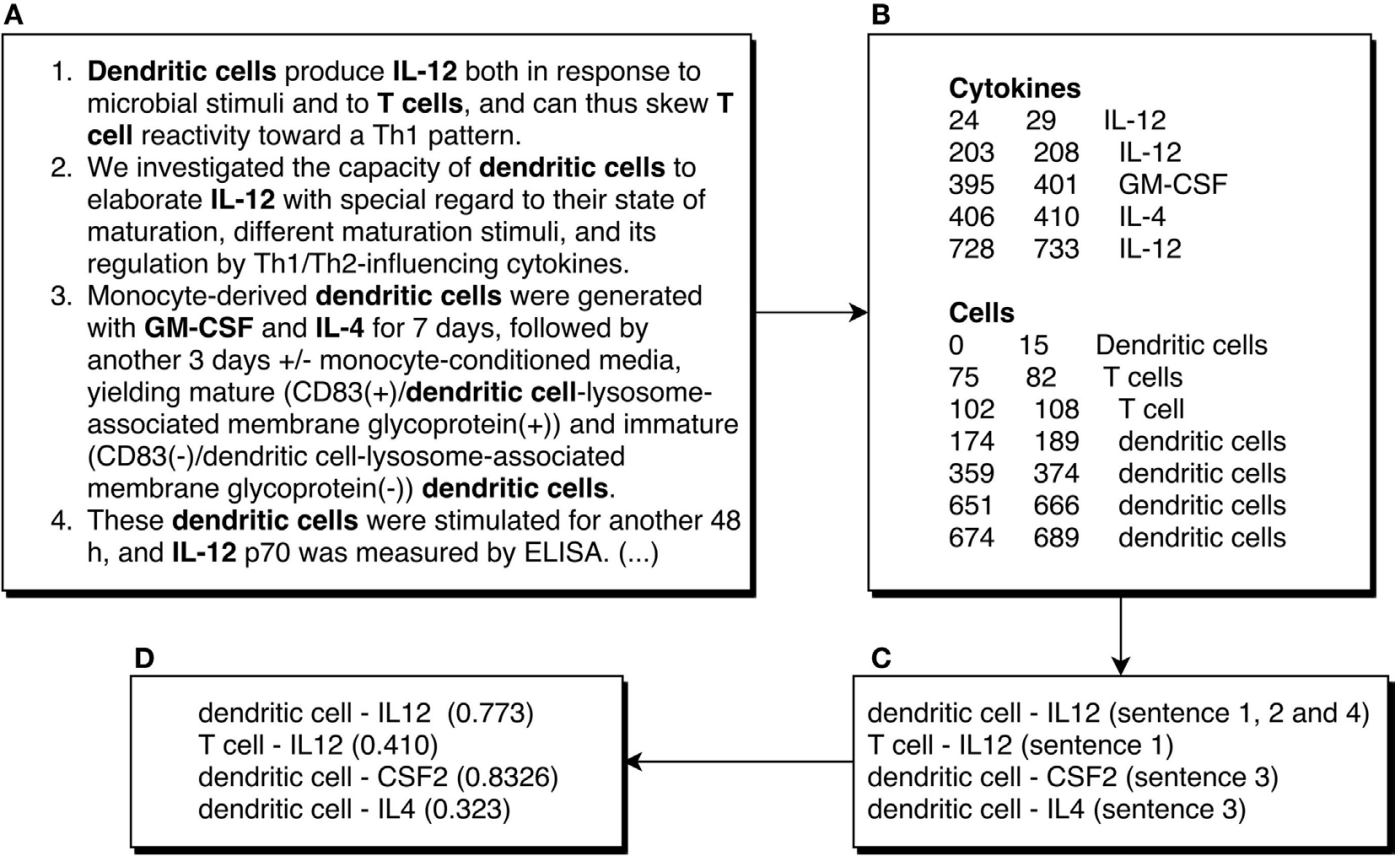
Example of an abstract being processed by the ICRel system. We show the first four sentences of the abstract of the article (12). The first box **(A)** shows these sentences, numbered and with cells and cytokines bolded manually. The second box **(B)** shows the entities recognized automatically, where the numbers at the start of each line represent the first and last character offset of the entity. The third box **(C)** shows the possible cell–cytokine combinations using the sentences shown. The fourth box **(D)** shows the confidence scores obtained with our system for those pairs. It should be noted that those scores were obtained using several documents and not just the example shown.

The objective of ICRel is to automatically generate a knowledge graph relevant to tolerogenic cell therapy from a given corpus. The system was written in Python 3.5 and its code is openly available.^3^ The methodology used can be adapted to other domains, by selecting an appropriate set of documents and reference database. Figure 1 presents the pipeline of ICRel, describing the input and output of each module, whereas Figure 2 provides an example of an abstract being processed by each module. The first module retrieves abstracts from PubMed into an internal database, according to a given query specified as input. The second module identifies named entities with an external tool, requiring one lexicon for each entity type to be identified. In this case, we had a lexicon for cell names and another for cytokines. The third module combines all cell–cytokine pairs identified within a sentence to generate instances for the machine learning classifier and to calculate the pair frequency score. Finally, the fourth module classifies each pair, assigns a confidence score and generates a graph based on the pairs that were classified as positive. The remainder of this section describes in detail the data and methods used to develop this system.

### 2.1 Datasets

A previous study provided a database of interactions between cytokines and cells, named immuneXpresso (13). Although this database was generated using automatic information extraction methods, its contents were evaluated with two manually curated databases, regarding the interactions containing B cells. The authors obtained a 20% false negative rate and no false positives. Even though we have no other guarantee that all entries of this database are correct, we considered this database as a silver standard due to the evaluation scores reported by the authors. A gold standard would require each entry to be manually validated by different domain experts. Since we could not find a gold standard for cytokine–cell interactions in abstracts, we used this silver standard to train and evaluate our method using 5-fold cross-validation. In previous studies, this type of methodology has been shown to be useful for information extraction evaluations (14, 15).

Each entry of the immuneXpresso database represents an interaction between a cytokine and a cell found in the literature. The interactions are supported by one or more abstracts, and they have the following attributes: direction (cell to cytokine or vice-versa), sentiment (Positive, Negative or Unknown), number of articles, and e-score. The sentiment reflects if the interaction indicates upregulation (positive) or downregulation (negative). Each interaction can be found in the associated abstracts, in at least one sentence mentioning both the cytokine and cell. We retrieved these abstracts from PubMed and associated each entry with the respective abstracts. A total of 25,347 abstracts were considered for this silver standard.

Our main objective was to develop an automatic system to generate a knowledge graph about cellular tolerogenic therapies, focusing on those that use APCs. Hence, we retrieved a corpus of documents related to this topic using the MeSH term “Antigen-Presenting Cells,” which should include most published abstracts with information relevant to our graph. We restricted this query to abstracts published from January 2015 to August 2017, to avoid overlapping with immuneXpresso, which has no abstracts published after 2015. Using this query, we obtained 3,264 abstracts, which were then annotated with cytokine and cell named entities. Figure 2A shows an excerpt of one of these abstracts. We expect that the information obtained by our system can be complementary to this database, which is not focused on any specific topic besides immunology. Furthermore, our system can automatically process new abstracts and add new relations to the graph.

### 2.2 Named Entity Recognition

Each abstract of our datasets contained named entities corresponding to concepts relevant to tolerogenic cell therapies. We were interested specifically in references to cells and cytokines in these abstracts. To this end, we established a lexicon of cell and cytokine names. The cell lexicon is based on the Cell Ontology (16) (version: 2017-07-29). We compiled all the concept labels and corresponding synonyms, resulting in a total of 8,503 terms. For cytokines, we used a cytokine registry,^4^ which includes several synonyms for each cytokine, corresponding to a total of 7,242 terms (version: November 2015). In both cases, each synonym was mapped to a reference string: Cell Ontology concept label in the case of cells and Entrez name in the case of cytokines. This way, we could associate the same entities mentioned across various documents through different synonyms, as long as those synonyms were considered in our lexicon.

We employed MER (17) to identify named entities in the abstracts. MER matches a list of terms (lexicon) to their mentions in the text, returning the characters of the entities found. For example, in the sentence “The dendritic cells were safely tolerated.” MER would return the characters from 4 to 19, which correspond to the text “dendritic cells.” Figure 2B shows an example of the output of MER for an abstract. This tool has the advantage of being easy to adapt to any entity type, it does not require annotated training data, and it is lightweight in terms of computational resources. We ran MER for each entity type (cell and cytokine) on each abstract. Due to its simplicity, MER has some limitations, for example, it is not able to use context to recognize entities, and it is susceptible to orthographic variations. To increase the number of entities recognized, we added plural variants of every cell name to the lexicon with the Python package inflect. This way, in the previous example, “dendritic cells” would be matched to the “dendritic cell” concept of the Cell Ontology, even if the text is not a perfect match. Furthermore, we removed common words such as “light” and “killer” from the cytokine lexicon, since these words could also appear in other contexts, for example, as part of “natural killer cell.” We found these words by comparing the lexicon to a list of common English words. The main limitation of MER is that the lexicon may be incomplete and some references to cells and cytokines in the documents will be missed. However, by using a large corpus, our assumption is that only rare variants will not be identified since most journals recommend a specific nomenclature for cells and proteins.

### 2.3 Cell–Cytokine Relation Extraction

**Figure 3:**
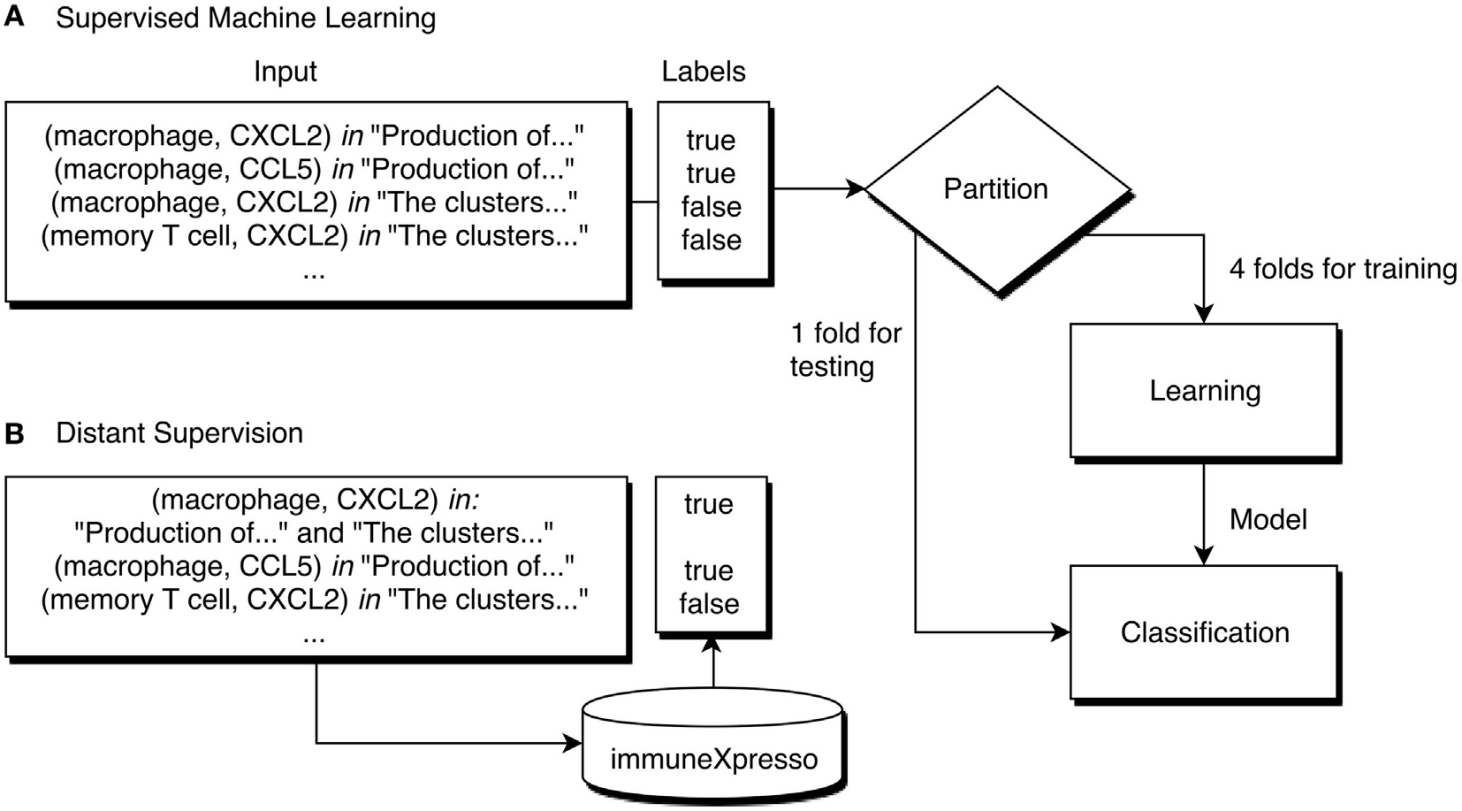
Demonstration of a machine learning workflow for cell–cytokine pair classification. **(A)** The label of each pair is known, and the learning algorithm trains a classifier based on these labels. Using 5-fold cross-validation, at each iteration 4 folds are used for training and 1 for testing. **(B)** Using distant supervision, the labels of each instance are not known, instead, a database assigns a label according to the existence of an entry corresponding to that pair.

A classifier is a model capable of assigning labels to new data according to a specific function learned from the training data. Supervised machine learning algorithms learn to classify instances (in this case, pairs) by adjusting a function to the labels of each instance of the training set. Generally, these algorithms require the training data to consist of a matrix where each line corresponds to an instance and each column to a feature. We consider an instance to be a specific combination of cell and cytokine, while the features consist of the words used in sentences where that pair cooccurs. A classifier should be evaluated to understand how useful it can be to predict the labels of new data. This type of evaluation is done by comparing the real labels assigned by experts to the labels predicted by the classifier. Figure 3A shows the workflow of the training and evaluation process of a supervised machine learning approach using 5-fold cross-validation. Cross-validation consists of iteratively partitioning the dataset in folds, using all but one of the folds to train a classifier. This classifier is used to predict labels for the remaining fold, which are then compared to the original labels. In a 5-fold cross-validation, this process is repeated 5 times, and an average of the scores obtained in each iteration is used to estimate the quality of the classifier. Afterward, a classifier can be trained using the whole dataset.

We consider a knowledge graph to be a set of facts associated with a specific domain using the RDF data model, i.e., specified by predicate–verb–object triplets. In our case, the knowledge graph is constituted by cell–cytokine interactions, where the focus is on the predicate and objects, which are cells and cytokines, with no specific order. An instance is any cooccurrence of a specific cell–cytokine pair within a sentence. We consider various types of relations, where a cell expresses a cytokine, or a cytokine affects the behavior of a cell. We are interested only in direct relations, where there are no intermediaries to the relation described. This includes cases of up- and downregulation, signaling, activation, and stimulation, for example. However, we are not interested in cases where the relation is negated (e.g., the cell does not express the cytokine) or hypothetical (e.g., the authors consider that a similar cell may express the same cytokine). For each pair, at least one sentence must explicitly state the existence of the relation for it to be considered a positive instance. That sentence may contain other information, such as the mechanism of the relation, experimental details or other cells and cytokines.

Distant supervision assumes that if a relation between two entities is stated in a database, it can be assumed that whenever those two entities cooccur in a document a relation between them is described (Figure 3B). We used distant supervision to generate a dataset for training since it is not easy to obtain labeled training data for most domains. For example, it would be assumed that every sentence in the abstract of the article (12) that mentions both dendritic cells and IL-12 is supporting that relation, including this sentence: “These dendritic cells were stimulated for another 48 h, and IL-12 p70 was measured by ELISA.” Although this assumption does not take into account the semantics of the text, it has been shown that distant supervision can be useful to extract relations from documents (18). In this work, we adopted immuneXpresso as the reference database. As previously mentioned, this database was generated automatically, however, the authors report a high accuracy when compared to experimental data.

The machine learning algorithm used by ICRel, multi-instance learning (MIL), organizes instances in bags, which consist simply of sets of instances with a common property. All instances are negative if the bag label is negative, or at least one instance is positive if the bag label is positive. Therefore, there is no need to manually label the relations in the documents. This approach can be applied to relation extraction, assuming that the instances are potential relations and the bags contain instances of the same pair of entities. Figure 2C shows an example of the way the instances are organized in bags, where each line corresponds to a different bag. Each bag has a label, which can be positive if the database contains an entry establishing a relation between the two elements of the bag, or negative otherwise. Using a machine learning algorithm, a classifier can be trained to classify new instances. This classifier will assign a confidence score to each bag. It is a reasonable assumption that an interaction is stated in a single sentence, so we consider only pairs of entities mentioned within a sentence.

Besides the labels of each bag, the MIL algorithm uses a feature representation of each instance to train a classifier. In our case, the feature representation of each instance is based on a window of words around each entity of the pair. We used a context window of size three, meaning that at most three words before and after each entity were considered. Each word was represented by its lemma so that variations of the same root word did not affect the learning process. Words that were part of named entities were represented by their respective entity type, to avoid any bias toward specific entities, and words that appeared in less than 1% of the documents were not considered, to reduced noise caused by text artifacts.

Then, we generated tf-idf weights for each word, to obtain a vector representation of each instance. Tf-idf corresponds to the product between term frequency (tf) and inverse document frequency (idf), and it is used to estimate the relative importance of each word in a corpus. This is required since machine learning algorithms require numeric vectors. The weights generated during the training phase were also applied to new data. In summary, each document was converted to sets of instances (bags), with each instance corresponding to a feature vector obtained with tf-idf weighting.

We observed that only some sentences in each abstract described relations between cells and cytokines, while the other sentences presented other types of information, such as definitions or experimental parameters. This would be an issue to traditional approaches relation extraction because there is a larger proportion of negative pairs (no direct and explicit relation is described in the text) than positive. In our preliminary experiments, we found that often less than 10% of the pairs in a document are positive. Therefore, it was necessary to use an algorithm that takes into account the sparsity of the data. We tested variations of MIL and found that sparse MIL (sMIL) (19) provided the best results. This algorithm is based on support vector machines, with an adapted objective function to account for the reduced number of positive labels. This new cost function assumes that smaller positive bags are more informative, weighting the feature vector of each positive bag according to its number of instances.

Our system contains a classifier trained using all entries and documents from the immuneXpresso database, corresponding to about 25k abstracts, using the methods described above. ICRel extracts relations from documents by transforming the text into feature vectors and then applying this classifier. The trained classifier predicts the label of a bag but does not predict the individual label of its instances. This means that it is not possible to know the exact sentence where the interaction is described. However, this information is sufficient for our purposes, since we know that each extracted relation has at least one sentence supporting it.

We used two different measures to classify an instance: the confidence score assigned by the machine learning classifier, and the number of sentences associated with a pair, which we call the pair frequency. The classifier confidence score was based on the distance to the hyperplane given by the sMIL algorithm, as described in Ref. (20). The pair frequency was calculated as the number of abstracts where that pair cooccurs in a sentence divided by the total number of abstracts in the corpus. We expect that pairs mentioned in more documents are more likely to have been correctly identified. Both scores were used to study how precision and recall varies when using a threshold. As the threshold increases, recall should decrease while precision increases.

### 2.4 Knowledge Graph for Tolerogenic Cell Therapy

The proposed ICRel system can extract candidate entries to generate a cytokine-cell graph. Each candidate entry is supported by the sentences where it was found, a classifier confidence score and its frequency. Figure 2D shows an example of the final output of the ICRel system. Since each cell and cytokine entity was normalized to a reference database, we can associate relations described over many documents, even if the authors use various nomenclatures. Furthermore, since we used the Cell Ontology as the reference for cell names, its axioms can be explored to expand the graph.

To obtain a knowledge graph for tolerogenic cell therapy, we first obtained a set of 3,264 documents about APCs. This set of documents does not overlap with the documents used to train the classifier, which includes only documents published before 2015. The same documents should not be used for training and testing machine learning classifiers because the classifier will have a biased performance on the training documents, leading to an overestimation of the quality of the results. Instead, we can simply match the immuneXpresso relations with our graph to obtain more knowledge.

The extracted relations were imported to Cytoscape (21) to visualize the graph. The ICRel graph is an undirected bipartite graph where each edge corresponds to a cell–cytokine relation. We compared our graph to the one obtained with immuneXpresso, by considering it also as an undirected graph. We computed standard properties of the two graphs, such as diameter and center nodes, with the Python package NetworkX (22). Furthermore, since our system is focused on obtaining information about tolerogenic cell therapies, we explored the information contained by each graph relevant to this type of therapy.

We considered that a manual evaluation of the automatically generated knowledge graph was necessary to estimate the quality of the information. We sampled a set of 60 edges to be manually validated by three human curators. Each curator validated 30 edges, with a set of 15 edges common to all three, to calculate the interannotator agreement. Each curator accepted an edge if there was at least one sentence supporting it in the corpus, and rejected otherwise. We asked to classify the cause of each rejection to understand the sources of error of our graph. The interannotator agreement was measured using Fleiss’ kappa, an adaptation of Cohen’s kappa for multiple annotators (23). The classifications of the curators were used to estimate the accuracy of the graph.

## 3 Results

The silver standard described in Section 2.1 is composed of 25,347 abstracts and a total of 4,445 cell–cytokine relations, without considering direction or any other attribute. The silver standard did not contain any information about entities mentioned in the abstracts that did not participate in cell–cytokine relations. We identified 185,243 cells and 189,457 cytokines mentions in these abstracts, which we then used to extract relations using the distant supervision approach. Considering that only 26,357 cell and 25,946 cytokines mentions exist in the immuneXpresso database, we identified about seven times more entities. Notice that these numbers refer to total mentions, i.e., any cell or cytokine may be mentioned more than once across the abstracts. We obtained a precision of 0.366 and recall of 0.853 when comparing with this silver standard. We estimate that the low precision is due to entities that do not participate in interactions, and, as such, are not considered in the silver standard used. For our objective, it is more important to recognize most of the cell and cytokines mentioned in the abstracts because the relation classifier will train and identify new relations based on those entities. Therefore, a recall of 0.853 indicates that most of the cell and cytokine names were identified.

**Figure 4:**
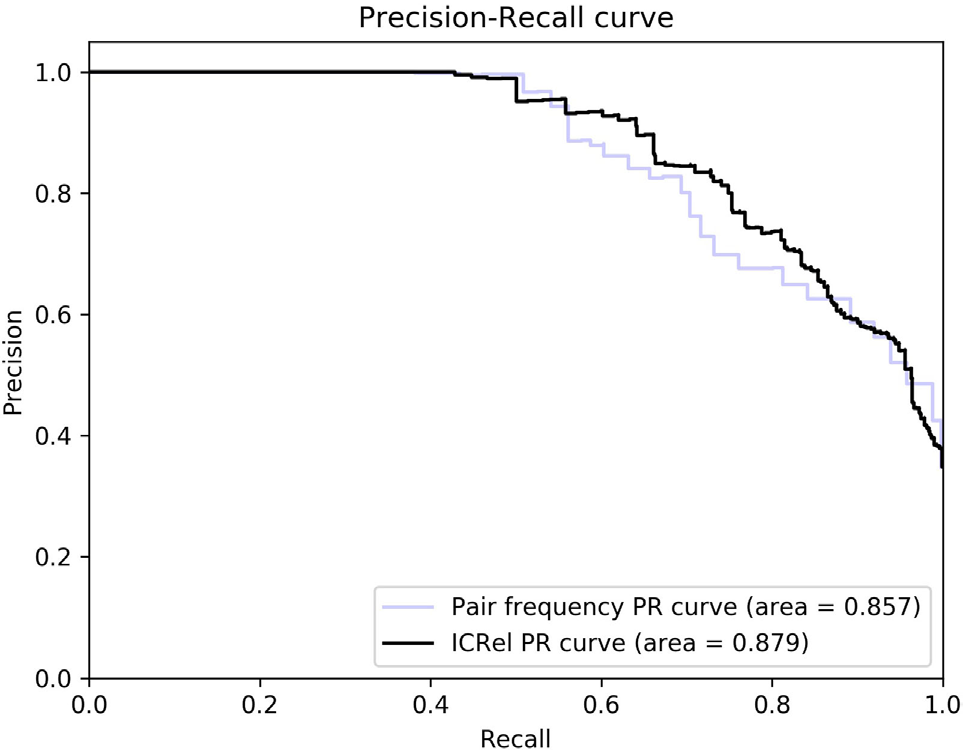
Precision-recall curves obtained using the classifier confidence score and pair frequency.

We ran a 5-fold cross-validation on the silver standard documents to evaluate the performance of our system. We randomly divided the documents into 5 partitions and iteratively trained a classifier on the documents and respective relations of 4 partitions and tested on the documents of the other one. Then we compared the relations obtained on each iteration with the silver standard, to calculate precision and recall. Using the classifier confidence score of each prediction, we can use it as a threshold to observe how it affects precision and recall. We compared this approach with only using the pair frequency, which was given by the number of documents where the cell and cytokine appeared within a sentence divided by the total number of documents. For both cases, we tested several threshold values and calculated precision, recall and F-measure assuming that only pairs with scores above the threshold were predicted as positive. Table 1 compares the confidence score calculated by the classifier with the pair frequency, at the threshold where the highest F-measure was obtained. Figure 4 shows the precision-recall curve obtained by ranking the pairs by classifier confidence or pair frequency. In this figure, we can see that for the same recall values, the distant supervision approach has higher precision than the frequency approach, hence it can provide higher quality results. At the highest recall values, the precision of the frequency approach is slightly higher, and for maximum recall, the precision is the same in both cases since the only difference is how the pairs are ranked. However, the classifier confidence score has a larger area under the curve (0.881 vs. 0.850). The area under the PR curve is used as an estimate of the quality of a classifier in cases where the distribution of the labels is skewed (24).

**Table 1:** Results obtained with cross-validation on the immuneXpresso silver standard using the classifier confidence score and pair frequency at the threshold where the highest F-measure was obtained.

	P	R	F1	Threshold
Pair frequency	0.753	0.718	0.735	0.126
ICRel	0.911	0.696	0.789	0.918

We generated a graph from the immuneXpresso database to compare with the graph generated using ICRel. This graph is composed of cell–cytokine relations found automatically in 25k abstracts from 1988 to 2015, resulting in 432 nodes and 2,495 edges. The authors of this database provided other properties for each relation, such as direction and degree. However, since our system did not provide this type of information, we considered all interactions regardless of their properties.

**Figure 5:**
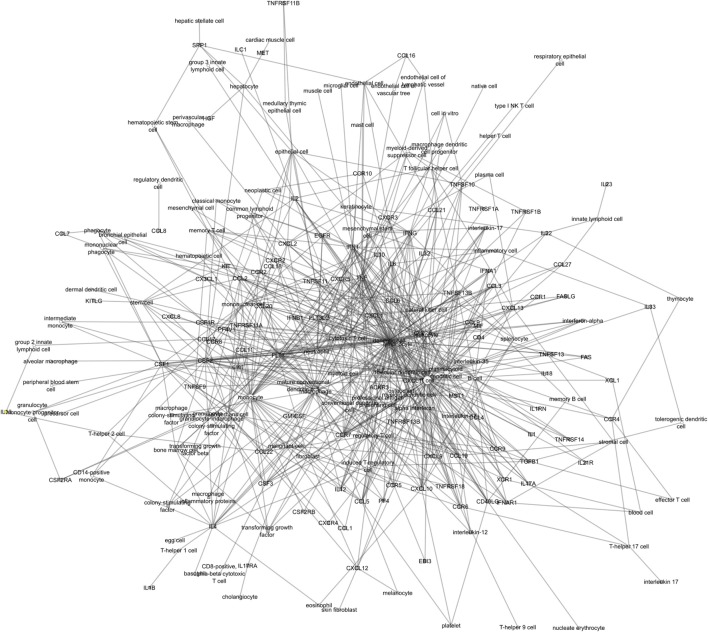
Overview of the ICRel knowledge graph. Cells are represented as white circles while cytokines are gray squares.

The ICRel graph contains 212 nodes and 647 edges, extracted from 3,264 abstracts. Each edge is supported by at least one sentence from these abstracts, with an average of 2.87 sentences per edge. Furthermore, each edge has a confidence value given by the classifier. We calculated the Pearson correlation between this confidence value and the number of sentences associated with the two nodes. We obtained a correlation of 0.666, which indicates that while the two variables are positively correlated, this correlation is not very strong. The diameter of this graph is 7, which is one edge larger than the immuneXpresso graph. Overall, the immuneXpresso graph contains more nodes and edges, which is expected since it was derived from a larger number of documents than the ICRel graph. Figure 5 presents an overview representation of the ICRel graph, while Table 2 provides a comparison between the two graphs. The files used to generated the graph are provided as supplementary material. Data Sheet 1 is a table where each line is an edge of the graph and the PubMed IDs of the documents are included, whereas Data Sheet 2 contains the sentences which support each of the edges.

**Table 2:** Comparison of ICRel and immuneXpresso graphs in terms of number of nodes, edges, abstracts used, and diameter.

	ICRel	ImmuneXpresso
Nodes	212	433
Cells	93	295
Cytokines	119	138
Edges	647	2,509
# abstracts	3,264	25,347
Diameter	7	6

Regarding the manual evaluation of the graph, the accuracy obtained was of 0.615. We obtained a kappa score of 0.600, which can be considered an adequate level of agreement (25). In the following section, we summarize the most common sources of error found in this evaluation.

## 4 Discussion

Our work demonstrates how text mining solutions can be used to automatically generate a knowledge graph relevant to tolerogenic cell therapy. A reference database is required to train a classifier based on a specific type of relation. Due to the lack of databases about immunological therapies, we could only train and evaluate our system on immuneXpresso. As such, we were also limited in terms of type of relation to extract, since it had to be a relation described in that database. However, cytokines have been shown to be therapeutic agents in various diseases such as diabetes mellitus and multiple sclerosis. Cytokines also have important roles in the production of APCs (3). It is relevant to understand the relation described in the literature between cells and cytokine since these could suggest novel approaches to tolerogenic cell therapy. Our graph contains these relations and can be integrated with other sources of information through the unique identifiers provided by the Cell Ontology or Entrez databases.

We compared the confidence score given by our classifier with a frequency-based approach, where the ranking score is given by the frequency of a cell–cytokine pair in the corpus. We found that the score given by the classifier is more accurate than the pair frequency. This is also supported by the low correlation between the classifier confidence and number of sentences supporting that pair (0.666). Our system learns how to classify relations using the context words as features. A cell–cytokine pair may be mentioned in multiple documents, but if the context words used are not similar to other positive pairs, it will not be classified as such. This is the main advantage of machine learning methods, along with the possibility of improving the classifier with more validated data.

Most of the processing time necessary to run our system consists of training the classifier. This part of the process takes more time and memory as more documents are considered for training since each document introduces new words and entities. In our case, the training itself took about 1 day. However, once the classifier is trained, a new set of documents can be processed relatively quickly.

### 4.1 Comparison between ICRel and immuneXpresso Graphs

The main point of comparison of our graph is the one created by Shen-Orr et al. (13), which we refer to as the immuneXpresso graph. This graph is larger than ours, containing more nodes and edges. However, it is important to consider that immuneXpresso was created using a more generic set of documents, that were retrieved using the keywords “Immunology and Allergy” and “General Science,” from a span of about 50 years. We demonstrated the usefulness of our system by generating a knowledge graph focused on one particular subject and using only abstracts published in the past two years. We expect that the number of relations extracted by our system would increase with a larger set of documents. Our assumption is that a more limited and focused set of documents should result in a graph with more relevant information to the subject of study.

**Figure 6:**
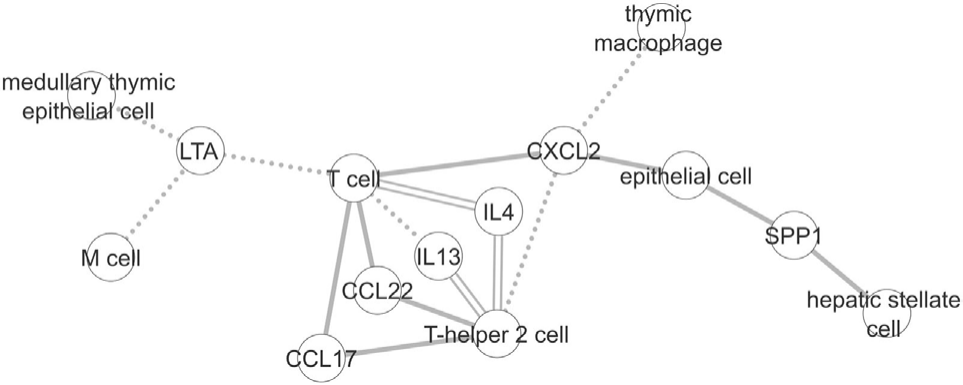
Subgraph created using the longest paths of the ICRel and immuneXpresso graphs with at least three nodes in common. Solid line corresponds to the edges of the ICRel graph, dashed line to the immuneXpresso graph, and double line to both.

We first compared the information stored in each graph in general terms. As shown in the Section 3, despite the difference in size, both graphs have a similar diameter. The diameter corresponds to the shortest distance between the two most distant nodes of a graph. As an example, Figure 6 shows a subgraph containing the union of the longest paths of each graph with at least three nodes in common. There are three edges in this subgraph that are shared between the two graphs (T cell < - > IL4, IL4 < - > T-helper 2 cell and T-helper 2 cell < - > IL13). These associations that exist in both graphs show that ICRel can extract well studied cell–cytokines relations, while in Section 4.2 we show examples of extracted relations from recent articles that could not be found in the immuneXpresso graph.

Comparing the relations described by each graph, we can observe various differences. The nodes in the center of the immuneXpresso graph (the center is the set of nodes whose distance to any other node is less or equal to the radius) are all cytokines (TGFB and TNG) while the ICRel graph has two cytokines (IL-6 and CSF2) and two cells (dendritic cell and T-cell) in the center. Dendritic cells are APCs, while T-cells can be targeted by APCs. Both cytokines CSF2 and IL-6 are also relevant to APCs since the former is used to differentiate APCs and the latter is produced by dendritic cells.

To better understand the degree of novelty of ICRel we divided its edges in four categories: (i) edges in common with the immuneXpresso graph; (ii) edges where the nodes existed in the immuneXpresso graph but were not connected; (iii) edges containing only one node that existed in the immuneXpresso graph; and (iv) edges where the two nodes did not exist in the immuneXpresso graph. Table 3 shows the total of edges for each of these categories.

**Table 3:** Degree of novelty of ICRel vs. immuneXpresso.

Category of edge	#
Present in both graphs	195
Unique to ICRel w/common nodes	178
Unique to ICRel w/a unique node to ICRel	256
Unique to ICRel w/both nodes unique to ICRel	18

Total	647

The two graphs have 132 nodes and 195 edges in common. The top five nodes that were in these edges were T cells (36), macrophages (20), TNF (19), CSF2 (17), and dendritic cells (15). Considering only nodes that were common to both graphs, ICRel found 178 new relations. For example, ICRel identified a relation between mononuclear cells and CSF2, supported by six documents.

The ICRel graph has 76 nodes (23 cells, 53 cytokines) that were not in the other graph. Of the new cytokines identified, most were actually genes coding cytokine receptors. However, we believe that these are as relevant to understand cell–cytokine relations as the cytokines themselves. A cell that produces a cytokine receptor is intrinsically associated with that cytokine. We found that 14 of the 76 new nodes were actually in the immuneXpresso database under different synonyms. For example, we identified the expressions “alpha interferon” and “interferon-alpha,” but we were not able to associate with IFNA, which is how it is represented in immuneXpresso. These synonyms should be considered in future analysis to facilitate the integration of different knowledge graphs.

The ICRel graph contains 256 edges with one new node, and 18 where the two nodes were new. The top five nodes of this category were T cells (27), dendritic cells (25), FLT3 (16), CCR7 (16), and monocytes (16). While the immuneXpresso graph contained many edges with T cells and dendritic cells, ICRel identified even more cytokines related to those cells. The FLT3 receptor is associated with the differentiation of dendritic cells, which might explain why our graph contains more edges with this cytokine receptor. CCR7 is a cytokine receptor annotated with the Gene Ontology term “positive regulation of dendritic cell antigen processing and presentation,” which was recognized by our system due to an entry in the cytokine registry that we used.

### 4.2 Manual Evaluation

We manually evaluated a partition of the ICRel graph to understand how a classifier trained on the immuneXpresso dataset would perform on a different corpus. This evaluation was performed by three researchers, who we refer to as curators, who read the sentences associated with 60 relations and determined if the cell–cytokine relation was supported by the text. The curators were given the same description of what was considered a relation, similar to the one presented in Section 2.3. We observed that the curators did not agree in some cases, leading to an interannotator agreement of 0.600, based on 15 relations. Since this value represented only a moderate agreement, we analyzed the cases where the curators disagreed. Our system considered both cytokine and cytokine receptors, and it was not clear to the curators which one was relevant. For example, one of the sentences contained the following text: “Flt3 ligand (Flt3L)”; our system recognized both FLT3LG and FLT3 and as cytokines, while FLT3 is actually a cytokine receptor. It is reasonable to assume that a cell associated with FLT3LG is also associated with its receptor, however, since it is not explicitly stated in the sentence, it caused ambiguity among the curators.

The accuracy obtained with the manual evaluation of the graph was of 0.615. The most common errors were indirect relation between the cytokine and cell, i.e., whenever there is a third element that affects both cytokine and cell. For example, consider the pair (CXCL2, memory T cell) in the sentence “(…) perivascular macrophages that are activated by IL-1a produced by keratinocytes and dDCs that are attracted by these macrophages through **CXCL2** signaling, both of which are essential for the efficient activation of **memory T cells** *in situ*.” Although both elements of the pair are mentioned in the sentence, there is not a direct relation described, instead, they are both directly associated with keratinocytes and dDCs.

Another common source of error is the incorrect recognition of named entities, both cytokines and cells. For example, in every sentence mentioning “granulocyte macrophage colony-stimulating factor,” macrophage was recognized as a cell entity. The cytokine registry we used to generate a list of synonyms contained some entries that were too ambiguous to be used by our system, such as acronyms that correspond to normal words. Although we were able to remove most of these synonyms, some cytokine synonyms stayed in the lexicon and generated named entity recognition errors. This is the case of immunoglobulin M (IgM), which was recognized as CD40LG since IGM is a synonym of that cytokine.^5^ These errors are hard to prevent since it is not possible to have complete knowledge of which synonyms have multiple meanings. One possible solution to this problem consists in computing the semantic similarity of all entities of an abstract and using that value to exclude outliers. Assuming named entity recognition errors would have low similarity to the other entities, this method could improve the precision of our graph (26). In the previous example, we expect that immunoglobulin M and CD40LG would have low similarity to the other entities of that abstract.

To identify if the graph contains information relevant to APCs, we evaluated manually the edges containing the node “professional antigen-presenting cell.” In the ICRel graph, this node is connected to 10 nodes: CCL19, CCL21, CCL5, CCR7, CSF2, CXCL12, IFN1, IL12, TGFB1, and TNF. Two of these cytokines (CSF2 and IL12) also appear associated with APCs in immuneXpresso. The ICRel graph contains the more generic IFN1, which includes two cytokines that appear associated with APCs in immuneXpresso (IFNA and IFNG). We confirmed the relations between APCs and its respective cytokines in the articles from where they were extracted (Table 4). By carefully analyzing the articles or the sentences provided in the supplementary material Data Sheet 2, it is possible to obtain more details about these relations. For example, Bryce et al. (27) explain the roles of CCL19 and CCL21 in the migration of APCs to lymph nodes. Since our system identifies both cytokines and their receptors, it also identified a relation between CCR7, a chemokine receptor, and APCs. Even though CCR7 is associated with APCs, as explained in this article, it is out of the scope of the knowledge graph, which consists of cell–cytokines relations (28). show that CXCL12 and CCL5 are relevant to the recruitment of APCs in early vitiligo. Although this is not directly related to tolerogenic therapies, understanding the mechanisms of APCs in disease can lead to new methods to generate and modulate the action of these cells. Further improvements could be added to ICRel in order to extract other attributes of each relation, such as directionality, temporality and magnitude. For example, by adapting the methods that we recently developed to classify the type, polarity, degree and modality of clinical events (29).

**Table 4:** Cytokines and receptors identified by ICRel as being associated with APCs.

		ICRel		immuneXpresso	
Cell type	Reference	APC	DC	APC	DC
CCL19	(27)	•	•		•
CCL21	(27)	•			•
CCR7	(27)	•	•		
CCL5	(28)	•	•		
CXCL12	(28)	•			•
CSF2	(30)	•	•	•	•
IFN1	(31)	•	•		•
IL12	(32)	•	•	•	•
TGFB1	(33)	•			•
TNF	(34)	•	•		•

*The second column indicates the reference of the abstract where that relation was found. The third and fourth columns indicate if that cytokine was associated with APCs or dendritic cells in ICRel and immuneXpresso respectively*.

To understand whether our method was able to find relations that were not yet well studied, we compared the cytokines associated with APCs and dendritic cells on ICRel and immuneXpresso (Table 4). ImmuneXpresso was generated using abstracts up to 2015, excluding that year. Only 2 of the 10 cytokines from ICRel were also found in immuneXpresso. Seven cytokines were found to be associated with APCs in articles from recent years. One cytokine receptor (CCR7) was also found to be associated with APCs and dendritic cells by our system. Our system as able to correctly extract this new information and organize it in a knowledge graph. We also studied the edges containing the node “dendritic cell,” which is a type of professional APC. The ICRel graph contains 64 edges associated with dendritic cells, of which 49 were not found in immuneXpresso. Dendritic cells and APCs had 7 edges in common in the ICRel graph (IFN1, CCR7, IL12, CSF2, TNF, CCL5, and CCL19). Comparing to the immuneXpresso graph, we can see that most of the cytokines associated with dendritic cells were found to be associated with APCs by ICRel. Since there is no overlap in the source documents, this means that while these cytokines were first reported to be associated with dendritic cells, other APCs types have also been studied, such as epidermal Langerhans cells (27) and macrophages (33).

We found that immuneXpresso lacked information about specific tolerogenic cell types, given that the version of the Cell Ontology used did not contain them. Thus, we added a list of 13 tolerogenic APC types to the lexicon so that relations containing these cells could also be detected. This led to the identification of 8 relations containing tolerogenic APCs (Table 5). The majority of these relations included myeloid-derived suppressor cells (MDSC). The system identified relations between MDSC and TNF, TNFRSF1A, and TNFRSF1B. While TNFRSF1A and TNFRSF1B are actually cytokine receptors, the article that mentions them (source article) describes the effects of gene deletion of both the cytokine and the receptors in carcinogenesis (35). The relation between MDSC and IL10 was extracted from a review article about the role of these cells in inflammatory diseases (36). Another relation extracted was between tolerogenic dendritic cells and TGFB1. In this case, the source article establishes the importance of TGFB1 in immunotherapies using tolerogenic dendritic cells (37).

**Table 5:** Relations of tolerogenic APC types found by the ICRel system.

Cell	Cytokine	Reference
Tolerogenic dendritic cell	TGFB1	(37)
Tolerogenic dendritic cell	IL33	(38)
Regulatory dendritic cell	CCL8	(39)
Myeloid-derived suppressor cell	TNF	(35)
Myeloid-derived suppressor cell	TNFRSF1B	(35)
Myeloid-derived suppressor cell	TNFRSF1A	(35)
Myeloid-derived suppressor cell	CXCL2	(40)
Myeloid-derived suppressor cell	IL10	(36)

### 4.3 Conclusion and Future Directions

Due to its initial stage, there is a lack of openly available databases about tolerogenic cell therapy. Although commercial databases such as COPE and Cytokine Reference exist, these depend on manual curation. It is time-consuming to manually develop and then update databases with newly found information from published articles. Our ICRel system presents a solution to this issue, by using machine learning to automatically generate a knowledge graph of cell–cytokine relations. Using the knowledge graph, experts can then find more facts to store in their own databases, or help them formulate new hypotheses that need further study. Our system obtained higher precision values when compared to a frequency based approach.

We demonstrated the usefulness of the system by focusing on antigen presenting cells relevant to tolerogenic cell therapy. There have been various advancements in our understanding of immune mechanisms and pathways that are dysregulated in autoimmune diseases, and active in transplant rejection, contributing to advancements in tolerogenic therapies. A better organization of the current knowledge about this process would benefit the development of new treatments and clinical trials. The knowledge graph contained relations between APCs that were found only in recent articles, thus showing how our system can lead to a more complete information structure on this topic. Furthermore, we identified multiple associations between specific tolerogenic APCs and cytokines. We believe that our proposed system has a large potential to help practicing cell biologists or cell therapy experts in identifying relevant relationships that can only be found by exploring various scientific articles in an integrated way. It was not our aim to find novel or specialized information but rather show the feasibility of the system and to use examples for guiding practitioners and experts on how to take advantage of it.

The work presented in this manuscript has two major applications. The first is information retrieval systems that can use the information from our graph to integrate various sources of information. This is the case of Bio2RDF (8), which stores several biomedical databases, such as KEGG, PubMed, and HGNC, in RDF format. The Bio2RDF project is an effort to link the entries of these databases using normalized URIs. Since our system matches each cytokine to the Entrez database and each cell to the Cell Ontology, it should be simple to integrate our graph with other databases for information retrieval. Another major application is recommendation systems. It is useful for a researcher working with a specific group of cell lines to know which other cells could also fit in that group. There are various methods to provide this type of recommendation, one of them consisting in exploring the structure of the graph to compute similarity measures. A recommender system could then suggest cells that interact with the same cytokines as the cells in the group. By integrating with external sources, it would be possible to suggest cytokines associated with specific diseases, chemicals or genes.

## Author Contributions

Conceptualization and methodology: AL and FC. Funding acquisition, project administration, and supervision: LC and FC. Investigation, validation, writing, review, and editing: AL, JF, LC, and FC. Software: AL. Visualization and writing original draft: AL, LC, and FC.

## Conflict of Interest Statement

The authors declare that the research was conducted in the absence of any commercial or financial relationships that could be construed as a potential conflict of interest. The reviewer PL and handling Editor declared their shared affiliation.

## Footnotes

^1^http://www.cells-talk.com

^2^https://www.google.com/intl/es419/insidesearch/features/search/knowledge.html.

^3^https://github.com/lasigeBioTM/ICRel.

^4^http://immport.org/immport-open/public/reference/cytokineRegistry.

^5^https://www.ncbi.nlm.nih.gov/gene/959.
